# Histopathological Classification—A Prognostic Tool for Rapidly Progressive Glomerulonephritis

**DOI:** 10.3390/medicina54020017

**Published:** 2018-04-17

**Authors:** Marta Kantauskaitė, Agnė Laučytė-Cibulskienė, Marius Miglinas

**Affiliations:** 1 Centre of Nephrology, Vilnius University Hospital Santaros Klinikos, LT–08661 Vilnius, Lithuania; agne.laucyte@gmail.com (A.L.-C.); marius.miglinas@santa.lt (M.M.); 2 Faculty of Medicine, Vilnius University, LT–03101 Vilnius, Lithuania

**Keywords:** rapidly progressive glomerulonephritis, crescentic glomerulonephritis, ANCA associated glomerulonephritis, histopathological classification, renal outcome

## Abstract

*Background*: Recently proposed histopathological classification may predict patient outcome in pauci-immune glomerulonephritis. This study sought to prove that the prognostic effect could be extended to all types of rapidly progressive glomerulonephritis. *Methods*: Retrospective analysis of patients diagnosed with rapidly progressive glomerulonephritis between April 1999 and August 2015 was performed. Epidemiological and clinical data were collected from medical records. The descriptions of renal biopsies were reviewed and classified into focal, sclerotic, crescentic and mixed class according to classification proposed by Berden et al. The study end points were end stage renal disease (ESRD) or death. Survival analyses were modelled using Cox regression. *Results*: 73 renal biopsies with diagnosis of rapidly progressive glomerulonephritis were included in the study. 25 (34.2%), 16 (21.9%), 24 (32.9%) and 8 (11%) patients were assigned to focal, crescentic, mixed and sclerotic class, respectively. Thirty-two (42.5%) patients were anti-neutrophil cytoplasmic antibody (ANCA) negative, of which eight (10.9%) were anti–glomerular basement membrane antibody (anti–GBM) positive and 24 (32.8%) were negative for autoimmune antibodies. Six (8.2%) patients died within one year. Among patients who survived, median change in estimated glomerular filtration rate (eGFR) values were: −10.5 mL/min in focal, 4.2 mL/min in crescentic, −4.3 mL/min in mixed and 4.1 mL/min in sclerotic group, *p* > 0.05. In the Cox regression model, there was no significant predictor of patient survival whereas the sclerotic group (HR 3.679, 95% CI, 1.164–11.628, *p* < 0.05) and baseline eGFR of <15 mL/min (HR 4.832, 95% CI, 1.55–15.08, *p* < 0.01) had an unfavorable effect for renal survival. *Conclusions*: Predominant glomerular sclerosis and low eGFR at baseline are associated with higher risk of ESRD in cases with crescentic glomerulonephritis. Therefore, despite the origin of injury, histological classification might aid in prediction of patient outcomes in rapidly progressive glomerulonephritis.

## 1. Introduction

Rapidly progressive glomerulonephritis (RPGN) is characterized by rapid decline in renal function and urinary abnormalities. It is a life-threatening syndrome which carries a risk of kidney failure requiring renal replacement therapy in up to 30% of cases [1,2]. Although it may look like a single entity, RPGN is a very heterogeneous disease. Indeed, the diverse etiology causes injuries within glomeruli trough alteration in various inflammatory pathways [3]. However, the processes initiating inflammation and destruction within the renal tissue underlie the same later occurring changes in glomeruli. Therefore, the tendency towards crescent formation and its distribution is likely to be more important for the evaluation and management of this disease [4,5]. 

RPGN results in patterns that could be identified using a light microscope and further characterized using other histological techniques. Therefore, currently it is classified into three types according to predominant immunological injury pattern: pauci-immune, immune complex or anti-glomerular basement membrane antibody mediated, respectively [6,7]. 

However, long-term insights into the prognosis of rapidly progressive glomerulonephritis imposed a formation of new look [8]. From the observation that different types of crescent forming glomerulonephritis have heterogeneous clinical outcomes, it evolved to histopathological classification [9,10,11,12]. The aim of the classification proposed by Berden was to simplify the diagnosis of pauci-immune glomerulonephritis while at the same time informing a clinician of the possible requirement for treatment adjustment. As the initial validation indicated that the sclerotic histological group had the worst treatment response, renal and patient survival chances, the question was raised if these patients are in need of an aggressive immunosuppression routinely used for rapidly progressive glomerulonephritis [12]. Immunosuppression is associated with an altered immune response and increased risk of infections as well as other complications leading to higher mortality rates [13,14,15].

This was the first step towards an individualized treatment approach. However, we still lack the information on which disease entities can be classified and which treatment rationales are the best for a given case. Therefore, further and extensive validation of histopathological classification is needed. Thus, we sought to apply this classification to a much broader spectrum than pauci-immune glomerulonephritis and to verify its power as a prognostic tool for different types of RPGN.

## 2. Materials and Methods

### 2.1. Study Design

For this study, patients with biopsies with rapidly progressive glomerulonephritis in Vilnius University Hospital Santaros clinics between 1999 and 2015 were retrospectively viewed. The syndrome of rapidly progressive glomerulonephritis was defined as a rapidly declining renal function (>50% within 3 months), oliguria and heamaturia and/or proteinuria in urine sediment. Patients’ demographics, clinical presentation and the course of disease, timing of renal biopsy, blood tests and treatment regimens were pooled from clinical records. After applying exclusion criteria (patient younger than 18 years old, follow up shorter than 6 months except for those cases when a patient was lost due to the death from renal disease) the final study was formed. The research has been approved by Vilnius Regional Biomedical Research Ethics Committee (No. 158200-14-744-261). 

### 2.2. Evaluation of Clinical Data

The presence of following extra-renal manifestation of glomerulonephritis was assessed: signs of lung or upper airways involvement, gastrointestinal symptoms, skin lesions, changes in vision or eye adnexa injury as well as joint damage. Constitutional symptoms were defined as fever, nausea, weight loss and/or myalgia. Blood count and inflammation markers were included into the study. ANCA and antinuclear antibody (ANA) testing were done by indirect immunofluorescence assay, anti—GMB positivity was assessed using the ELISA. Peak serum creatinine level at the admission to hospital and at 3 month interval during follow up was recorded. Glomerular filtration rate was estimated using four-variable Modification of Diet in Renal Disease (MDRD) formula.

### 2.3. Histopathology

Renal biopsy specimens were prepared using standard techniques for light microscopy. As described by Berden, the following glomerular lesions were evaluated: (i) normal glomeruli defined by minimal signs of inflammation and ischemia without vasculitis or sclerosis, (ii) crescents—either purely or partially cellular in which extracellular matrix accounted for <90%, (iii) global sclerosis—sclerotic changes in glomerular tuft >80%. According to the occurrence of the aforementioned lesions, the biopsies were assigned to one of four groups: focal, crescentic, mixed or sclerotic. The focal category was defined as >50% normal glomeruli in renal biopsy, crescentic as >50% glomeruli with crescents, sclerotic as >50% global sclerosis in glomeruli and, finally, the mixed category included combination of changes described other groups. Adequate biopsy must contain at least 10 glomeruli. Interstitial and tubular lesions were scored according to percentage of compartment that was affected: interstitial infiltration (−, 0%; +, 0–50%; ++, >50%), interstitial fibrosis (−, 0%; +, 0–50%; ++, >50%), tubular atrophy (−, 0%; +, 0–50%; ++, >50%) and tubular necrosis (−, 0%; +, 0–100%).

### 2.4. Treatment

The induction therapy of pauci-immune and anti-GBM glomerulonephritis included combination of cyclophosphamide and corticosteroids. Cyclophosphamide was given intravenously with dose varying 0.5–1 mg/m^2^ monthly, or oral 2 mg/kg daily up to 6 months. Before the first cyclophosphamide pulse patients were pretreated with 3 pulses of 1 g methylprednisolone and assigned to oral corticosteroids with a dose of 1 mg/kg daily which was gradually tapered after one month. Patients with pulmonary hemorrhage, which was confirmed by radiological tests, additionally, received in addition plasma exchange treatment with 60 mL/kg of volume replacement. In total, patients received 6 procedures which were performed daily or every other day depending on the severity of the disease. For maintenance either azathioprine (1.5–2 mg/kg) or methotrexate (0.3 mg/kg) or low dose corticosteroids (5–10 mg/d) were given. Patients with lupus nephritis received oral steroids 1 mg/kg which were tapered after 1 month of the treatment. The drug for maintenance treatment was chosen between azathioprine, methotrexate and low dose steroids. In the case of post-infectious glomerulonephritis the primary disease was treated.

### 2.5. Outcomes

The clinical endpoints were death related to kidney disease and end stage renal disease defined as a constant need of dialysis, either hemodialysis or peritoneal dialysis, or renal transplantation. Patients free from kidney disease were dialysis independent, who did not require immunosuppression, had creatinine concentration of normal range, proteinuria of <300 mg/24 h and no signs of hematuria in 24-h urine. All other cases were defined as responders-dialysis independent patients, showing low activity of kidney disease. Clinical endpoint was evaluated during the last follow up.

### 2.6. Statistical Analysis

Descriptive data are presented as mean ± standard deviation (SD) or median with interquartile range (IQR) values for continuous variables and as n (percentage) for categorical ones. One way ANOVA was used for variable comparisons when data was normally distributed or Kruskal-Wallis test in other cases. Chi-square test was used for dichotomous variables. Cox regression model was established to forecast patient and renal survival. *P*-values were two tailed and considered significant when <0.05. The software used for statistical analysis was SPSS 17.0 (IBM, Armonk, NY, USA).

## 3. Results

During the study period 86 cases with RPGN confirmed by renal biopsy were found. After application of exclusion criteria, 73 cases were further analyzed. Table 1 lists the demographics and baseline characteristics of study objects. Twenty-five (34.2%), 16 (21.9%), 24 (32.9%) and 8 (11%) patients were assigned to focal, crescentic, mixed and sclerotic class, respectively. There were 37 (50.7%) female and 36 (49.3%) male patients with the average age of 53.4 ± 16.8 years at the time of renal biopsy. Sex and age distribution among histological groups was similar. Average follow up period was 759 (474 to 1367) days with the shortest being 69 days and the longest being 5391 days. The most common injury pattern was pauci-immune glomerulonephritis, accounting for 63% of cases. Twenty-four (32.9%) patients were diagnosed with ANCA positive rapidly progressive glomerulonephritis, which was defined as renal limited vasculitis with positive ANCA test, and 12 (16.4%) with granulomatosis with polyangiitis (formerly known as Wegener’s granulomatosis). ANCA glomerulonephritis was more common clinical diagnosis among patients with mixed renal injury pattern, accounting for most of the cases. Other types of RPGN formed smaller parts of our population. For example, cases with anti-glomerular basement membrane antibody associated disease composed 8.2% and cases with immune complex mediated injury composed 27.4% of the study population.

There was pANCA predominance over cANCA among all histological groups. Eight patients were double positive for ANCA and ANA. ANCA negative patients made up the following histological subgroups: focal 48%, crescentic 37.5%, sclerotic 25% and mixed 33.3%. Among ANCA negative patients, other types of antibodies were observed. There were four patients with anti-GBM antibody positivity in focal histological group, two in the crescentic group and one patient in the sclerotic group. 

Constitutional symptoms were present in more than 90% of patients. Extra-renal organ damage was present in two thirds of cases except for the sclerotic group (37.5%). The most common organ target in this group was respiratory system. In focal, crescentic and mixed groups the damage to this system was present in *n* = 13 (52%), *n* = 8 (50%), and *n* = 7 (29.2%) patients, respectively. Joint damage was the second most commonly targeted organ system and was present in 20%, 25%, 25% and 12.5% in focal, crescentic, mixed and sclerotic groups, respectively.

In each renal sample an average of 18.5 ± 7.3 of glomeruli were seen. The lowest rate of normal glomeruli was seen in the mixed group, 22.5 ± 3.5% respectively (Table 2). Tubular atrophy defined as flattened as well as vacuolated tubular epithelial cells, was pronounced among all groups, except the crescentic group. In this group it was identified only in 6.3% of biopsies. The minimal level of inflammation (<50%) was predominant among all the histological groups—focal (*n* = 19; 79.2%), crescentic (*n* = 13; 81.2%), mixed (*n* = 22; 91.6%) and sclerotic (*n* = 7; 87.5%). There were only four cases free of inflammation, three from the focal and one from the mixed group. 

At the admission, patients from the mixed group had markedly higher levels of creatinine (median creatinine concentration was 374 µmol/L and eGFR 47.64 mL/min). During the first year, creatinine levels were the highest in the sclerotic group and the lowest in the focal and crescentic groups (Table 3). There were only five patients whose eGFR was above 90 mL/min, seven more patients had eGFR values between 8960 mL/min. During the second year of follow up, a slight shift was observed—kidney function in crescentic, mixed and sclerotic group deteriorated and improved in the focal group. 

Eighteen (24.7%) patients received oral cyclophosphamide and 55 (75.3%) patients received it intravenously. In addition, 10 patients (13.7%) received plasma exchange treatment (due to already diagnosed or high possibility of pulmonary haemorrhage). During the acute period haemodialysis was initiated in three (9.4%), five (29.4%), and two (14.3%) patients from the focal, crescentic, and mixed groups, respectively. All patients assigned to the sclerotic group were under haemodialysis during an acute disease stage. Maintenance therapy with azathioprine was applied to 15 patients (20.5%), methotrexate was received by two patients (2.7%), and the other patients received small doses of steroids. Five (6.8%) patients did not receive any type of immunosuppressive medication. 

During the study period, six deaths associated with renal disease and its complications were recorded. Three of those patients were from the focal group, one was from the crescentic group and two were from the mixed group. Those patients from the focal group were diagnosed with Goodpasture’s syndrome. Histological class, tubular injury, creatinine level, gender and ANCA positivity were not found to have an effect on patient survival (Table 4). ESRD incidence rates in focal, crescentic, mixed and sclerotic groups were 28%, 31.25%, 29.2%, 62.5%, respectively. Patients free from renal disease in each group comprised less than 20% of cases, except in the sclerotic group where no such cases were observed. Cox regression analysis for renal survival during the follow up period showed that the sclerotic group had the worst prognosis. However, there were no significant differences between the sclerotic and other histopathological groups. Other clinical or pathological criteria used to plot the regression model are listed in Table 4.

## 4. Discussion

The present study was carried out to assess the prognostic effect of histological classification proposed by Berden in all patients with rapidly progressive glomerulonephritis (RPGN). It is the first study where immunological injury pattern was not taken into account and the first from Lithuania and the Baltic states on outcomes of RPGN. Patients with ANCA positive glomerulonephritis made up 16.4% of our population, whereas cases other than pauci-immune glomerulonephritis made up 27% of the total cohort. In contrast to other validation studies, we present a wider spectrum of patients with biopsy confirmed RPGN. 

Almost half (49.3%) of the study population were ANCA negative. ANCA negative patients were mainly from the focal and crescentic groups (48% and 37.5%, respectively). It was reported previously that a pauci-immune glomerulonephritis with negative antibody test is associated with the crescentic group and with a worse renal outcome [16,17,18]. However, we have not found any association between ANCA negativity and an unfavourable patient or renal outcome.

In our study population, 34.2% of patients were assigned to the focal histological group. Similar predominance was seen in the validation study performed by Iwakiri [19]. Primary study representing the European population and some carried out later indicated the crescentic group as the dominant one [12,20]. The least common was the sclerotic group. We did not observe any cases with features specific to two or more histological groups. The proportions of normal, crescentic or sclerotic glomeruli among the groups were different than previously reported. The percentage of normal glomeruli was higher in the crescentic and sclerotic group compared with mixed. Other studies on histopathological classification identified the sclerotic group as having the lowest amount of normal glomeruli [12,21]. However, we believe that this result could be a consequence of traction of healthy renal tissue because of the sclerosis process. As for number of sclerotic glomeruli, the crescentic group had the lowest values. The same association was noticed by the other research [22]. Other authors were able to show the negative effect of tubular atrophy [9,23,24]. In the present study, neither tubular injury nor interstitial fibrosis was an indicative renal survival factor. However, there were some significant differences between the histological groups. Tubular injury was pronounced in all groups except for crescentic, whereas interstitial fibrosis was pronounced only in the sclerotic group. The presence of interstitial fibrosis in the sclerotic group confirms the hypothesis that the injury has already caused irreversible changes. The discrepancy between the percentage of tubular and interstitial injury among other groups might be associated with disease severity. Patients from the focal group were more often diagnosed with Goodpasture syndrome or granulomatosis with polyangiitis. These diseases are associated with rapid manifestation, renal and extra-renal symptoms, which might include hypovolemia and higher risk of renal tubule injury. Therefore, we believe that more severe disease and a shorter time to biopsy might have had an effect on the presence of tubular and interstitial injury.

In comparison with other validation studies, our patients had better renal function at the time of hospitalisation [22,25]. Regardless, there was a tendency towards ESRD progression, whereas other studies have observed significant improvement of renal function [25,26]. However, eGFR at presentation remained a strong prognostic factor for renal outcome. 

After performing Cox regression analysis for renal survival, we identified the sclerotic group as having the highest risk for ESRD in two years. These results are in accordance with the primary study and validation studies presenting Canadian, American, European, Japanese and Chinese populations [19,21,22,25,26,27,28]. Low baseline eGFR was associated with progression to ESRD as well, whereas tubular injury had no effect. 

In our study, deceased patients comprised 8.2% of cases. Other studies indicated such outcomes ranging up to 50% [29]. The more favourable outcome in our cohort might be related to early diagnosis of crescentic glomerulonephritis. However, we are not able to compare this aspect with other studies as they defined the time of biopsy as the time of disease presentation [30]. In addition, a large amount of patients (75.3%) received vigorous treatment with intravenous cyclophosphamide. The percentage distribution of death cases for focal, crescentic, mixed and sclerotic groups was as follows: 12%, 6.25%, 8% and 0%. Other validation studies do not indicate any death cases in the focal group. Our results were influenced by the fact that there was a high incidence of Goodpasture syndrome in the focal group which has long been known to carry a higher mortality risk. For these specific patients, even the detection of disease during an acute period and early treatment initiation was not effective.

The present study has an advantage of being a single-centre study with routinely performed renal biopsies and precise treatment protocols. In contrast to previously published validation studies, we have represented a RPGN population which is wider and more diverse as patients who were ANCA negative and who had diseases other than pauci-immune glomerulonephritis were included. However, the retrospective collection of all data limits its usefulness. Other limitations include small number of patients and uneqaul distribution of different immune patterns of RPGN. Statistical data was not constant due to the loss of patients during follow up and due to missing data.

## 5. Conclusions

Patients with extent sclerosis in their renal biopsy specimens and high baseline creatinine concentrations have a higher risk of developing end stage renal disease. This negative outcome is present despite treatment with aggressive immunosuppression. Such patients might have better prognosis and lower rates of adverse effects related to immunosuppression if treated conservatively. Of course, further clinical studies are needed to confirm our results and to move histopathological classification into daily protocols.

## Figures and Tables

**Table 1:** Clinical and biological characteristics of patients.

	Focal	Crescentic	Mixed	Sclerotic
N of patients	25	16	24	8
Male sex, *n* (%)	11 (44)	8 (50)	12 (50)	5 (62.5)
Age, mean ± SD, years	50.5 ± 20.4	55.7 ± 13.2	56.7 ± 15.5	48.2 ± 14.2
Follow, median (IQR), days	1334 (2283273)	748 (5111529)	813 (596923)	506 (468700)
Urea, median (IQR), mmol/L	16.3 (10.2–30.5)	11.6 (6.2–34.6)	19.6 (7.734.8)	17.3 (5.228)
CRP, median (IQR), mg/L	38 (10.4102.4)	15.5 (652.6)	67.9 (22144.7)	3.6 (0.8134)
Time to biopsy, median (IQR), days	13 (335)	6 (316)	10 (425)	7 (640)
Immunology, *n* (%)				
pANCA	8 (32)	8 (50)	10 (41.7)	3 (37.5)
cANCA	3 (12)	0 (0)	5 (20.8)	1 (12.5)
anti-GBM	4 (16)	2 (12.5)	0 (0)	1 (12.5)
ANA	2 (8)	2 (12.5)	2 (8.3)	2 (25)
No antibodies detected	8 (32)	4 (25)	8 (33.3)	1 (12.5)
Constitutional symptoms, *n* (%)	23 (92)	13 (92.9)	24 (100)	8 (100)
Extra-renal symptoms, *n* (%)	15 (60)	9 (56.3)	16 (66.7)	3 (37.5) *
Respiratory system	13 (52)	8 (50)	7 (29.2)	2 (25)
Joints	5 (20)	4 (25)	6 (25)	1 (12.5) *
Gastrointestinal system	0 (0)	0 (0)	1 (4.2)	0 (0)
Eyes	0 (0)	0 (0)	0 (0)	0 (0)
Skin	5 (20)	2 (12.5)	6 (25)	0 (0) *
Diagnosis, *n* (%)				
ANCA (+) glomerulonephritis	6 (24)	4 (25)	12 (50)	2 (25)
ANCA (−) glomerulonephritis	2 (8)	2 (12.5)	1 (4.2)	1 (12.5)
Goodpasture Syndrome	4 (16)	1 (6.3)	0 (0)	1 (12.5)
Granulomatosis with polyangiitis	5 (20)	3 (18.7)	2 (8.4)	2 (25)
Allergic granulomatous angiitis	1 (4)	0 (0)	0 (0)	1 (12.5)
Systemic lupus erytheamathosus	2 (8)	0 (0)	0 (0)	0 (0)
Microscopic polyangiitis	1 (4)	1 (6.3)	1 (4.2)	0 (0)
Post-infectious glomerulonephritis	4 (16)	5 (31.2)	8 (33.3)	1 (12.5)

Data are presented as mean ± SD or median (IQR) or number (%). * Other groups vs. sclerotic (chi-square test).

**Table 2:** Histopathological characteristics.

	Focal	Crescentic	Mixed	Sclerotic
Normal glomeruli	65.5 ± 4.9 *	43.7 ± 7.1	22.5 ± 3.5	41.6 ± 10.4
Cellular crescents	20.8 ± 3	73.7 ± 3.7 **	22.3 ± 2.2	36.6 ± 12.9
Global sclerosis	16.1 ± 4.2	11.6 ± 3.4	17.0 ± 2.8	71.7 ± 4.8 ^
No tubular damage	12 (48)	15 (93.8) **	15 (62.5)	3 (37.5)
Tubular atrophy <50%	1 (4)	0 (0)	3 (12.5)	2 (25) ^
Tubular atrophy >50%	12 (48)	1 (6.3)	6 (25)	3 (37.5)
Tubular necrosis	15 (46.9)	1 (6.3) **	7 (50)	4 (50)
No signs of inflammation	3 (12.5)	0 (0)	1 (4.2)	0 (0)
Inflammation >50%	2 (8.4)	3 (18.8)	1 (4.2)	1 (12.5)
Inflammation <50%	19 (79.2)	13 (81.2)	22 (91.6)	7 (87.5)
No signs of interstitial fibrosis	20 (80)	10 (62.5)	22 (91.6)	2 (25)
Interstitial fibrosis <50%	5 (20)	6 (27.5)	2 (8.4)	4 (50)
Interstitial fibrosis >50%	0 (0)	0 (0)	0 (0)	2 (25)^

Data are presented as mean ± SD or number (%). * Other groups vs focal (ANOVA; chi-square test); ** Other groups vs crescentic (ANOVA; chi-square test); ^ Other groups vs. sclerotic (ANOVA; chi-square test).

**Table 3:** Evolution of renal function.

	Focal	Crescentic	Mixed	Sclerotic
Creatinine at entry, μmol/L	227 (105 to 555)	158 (101 to 506)	374 (147 to 633)	280 (83 to 436)
Creatinine at 1 year, μmol/L	152 (93.5 to 450)	190 (143 to 378)	260 (104 to 627)	637 (268 to 750)
Creatinine at 2 years, μmol/L	196 (99 to 329) **	234 (115 to 547)	295 (113 to 540)	689 (450 to 776)
eGFR at entry, mL/min	41.3 (10.7 to 88.9)	27.86 (8.2 to 54.3)	47.64 (8.4 to 80.3)	10.16 (7 to 17.8) *
eGFR at 1 year, mL/min	37 (10.1 to 70.8)	24.5 (11.8 to 46.9)	19.4 (7.4 to 67.4)	7.7 (7.5 to 21.9)
Change vs. entry	−15.1 (−38.6 to 15.5)	5.6 (1.4 to 22.8)	−2.7 (−41 to 6.6)	−2.7 (−3.9 to 3.7)
eGFR at 2 years, mL/min	38 (11 to 49.6)	21.1 (19.5 to 38)	24.3 (15 to 50.4)	6.5 (8.2 to 29)
Change vs. entry	−10.5 (−33.5 to 10.2)	4.2 (−0.6 to 7.4)	−4.3 (−5 to 11.2)	4.1 (3.3 to 8.1)

Data are presented as median (IQR). * Other groups vs sclerotic (Kruskal–Wallis test); ** Focal vs. sclerotic (Kruskal–Wallis test). eGFR, estimated glomerular filtration rate. Modification of Diet in Renal Disease (MDRD) formula was used for eGFR estimation.

**Table 4:** Predictors of patient survival and end stage renal disease (ESRD) in Cox regression model.

	Mortality risk			ERSD risk		
	HR	(95% CI)	*p* value	HR	(95% CI)	*p* value
Histopathological group						
Focal	1			1		
Crescentic	0.497	0.052–4.783	0.56	1.018	0.323–3.209	0.98
Mixed	0.634	0.106–3.809	0.62	0.923	0.323–2.633	0.88
Sclerotic	0	0	0.99	3.679	1.164–11.628	0.03
Tubular injury						
Intact	1			1		
Atrophy	1.036	0.905–8.541	0.55	0.364	0.109–1.210	0.17
Necrosis	1.25	0.974–6.52	0.42	0.131	0.016–1.081	0.06
eGFR						
>60 mL/min	1			1		
30–59 mL/min	2.891	0.181–46.28	0.45	0.584	0.018–3.943	0.64
15–29 mL/min	3.407	0.213–54.47	0.39	0	0	0.98
<15 mL/min	4.016	0.411–39.26	0.23	4.832	1.55–15.08	0.01
ANCA antibody						
Yes	1			1		
No	1.449	0.254–8.321	0.68	0.814	0.267–2.484	0.7
Gender						
Male	1			1		
Female	0.861	0.332–10.45	0.48	0.626	0.245–1.597	0.32
Extra-renal symptoms						
Yes	1			1		
No	2.01	1.042–5.012	0.15	1.787	0.642–4.974	0.22
